# Using photovoice to explore young women’s experiences of behaviour change techniques in physical activity mobile apps

**DOI:** 10.1186/s12966-023-01447-9

**Published:** 2023-04-14

**Authors:** Mollie M. Tobin, Tamara L. Jones, Yui Sum Haylie Ho, Camille E. Short

**Affiliations:** 1 Melbourne Centre for Behaviour Change, Melbourne School of Psychological Sciences, University of Melbourne, Melbourne, VIC Australia; 2 Melbourne School of Health Sciences, University of Melbourne, Melbourne, VIC Australia

**Keywords:** Photovoice, Behaviour change, Physical activity, Digital health, Phone apps

## Abstract

**Background:**

Research shows that inactive young women are attracted to using mobile phone applications (apps) to increase physical activity. Apps can promote physical activity by delivering a range of behaviour change techniques to influence determinants of user behaviour. Previous qualitative research has examined user experiences with techniques in physical activity apps, however there is little research specifically among young women. This study aimed to explore young women’s experiences using commercial physical activity apps to change their behaviour.

**Methods:**

Young women were recruited online to use a randomly assigned app for two weeks to achieve a personal goal. Using photovoice, a qualitative participatory research method, participants generated insights about their experiences through photographs and semi-structured interviews. Thematic analysis was conducted on photograph and interview data.

**Results:**

Thirty-two female participants, aged 18–24 years, completed the study. Behaviour change techniques tended to cluster around four key themes: logging and monitoring physical activity; reminders and prompts; workout videos and written instructions; and social features. Social support also strongly influenced participants’ experiences.

**Conclusions:**

Results suggest that behaviour change techniques influenced physical activity in line with social cognitive models, and these models are useful to understand how apps can target user behaviour for young women. The findings identified factors important for young women that seemed to moderate their experiences, such as social norms about women’s appearance, which should be further explored within the context of behaviour change models and app design.

**Supplementary Information:**

The online version contains supplementary material available at 10.1186/s12966-023-01447-9.

## Background

Physical inactivity is a global health problem and a primary risk factor for non-communicable diseases, which now account for the majority of deaths worldwide [1, 2]. Large-scale research has shown that women are less physically active than men [3, 4], with this gender gap beginning in late adolescence and continuing throughout the lifespan, with women becoming more inactive as they age [5].

To support greater physical activity participation among young women, gender-specific research is required to understand how to better promote physical activity behaviour in this cohort [6]. From a public health perspective, large-scale, cost-effective physical activity promotion programs are critically needed [7]. Consequently, digital health interventions are being prioritised as tools that policymakers can use to target inactive populations, including girls and women [8].

With most adults now owning a smartphone, mobile phone applications (apps) are increasingly being used for physical activity promotion and have the potential to deliver wide-reaching interventions [9]. Apps can promote physical activity by delivering a range of strategies, or behaviour change techniques, that aim to influence determinants of user behaviour [10]. Systematic reviews and meta-analyses have shown that apps and mobile technologies can significantly impact physical activity behaviour [11, 12]. However, small effect sizes [13, 14] and high levels of non-usage are typically reported [15].

Compared to the broader population, inactive young women are reportedly more attracted to using physical activity apps [16], but stop using apps more often than men [17]. Therefore, whilst young women consider apps to be an acceptable physical activity intervention, apps may not be meeting their behaviour change needs. To design more effective interventions, research should seek to understand how young women experience behaviour change techniques embedded within apps.

Content analyses suggest that commercially available physical activity apps use between five to nine behaviour change techniques [9, 18, 19]. The most frequent techniques in top-ranked physical activity apps include providing feedback and monitoring of physical activity, encouraging goal setting, facilitating social comparison, and planning social support from friends and family [9, 18–20]. These techniques are also commonly used in physical activity apps designed and evaluated by researchers [21]. Whilst considerable research has now focused on how physical activity interventions work as a package, researchers understand less about how (and why) individual behaviour change techniques work to change behaviour within digital interventions, and particularly how they are experienced by young women.

To enhance physical activity app design for young women a clearer understanding of two processes is needed: 1) the process through which individual behaviour change techniques influence behaviour, and 2) the design elements of behaviour change techniques that contribute to engagement. Drawing on theories is recommended in both regards, as theories provide insight into the possible mechanisms of action through which a behaviour change technique or design element influences behaviour [22]. Selecting an appropriate theory and mapping behaviour change techniques to theoretical constructs and operationalising them in an engagement promoting way remains a significant challenge, however. There are multiple behaviour change techniques that can be used to target a single mechanisms of action [23]. Likewise, behaviour change techniques can influence multiple mechanisms at once. Design elements, such as the extent to which the content is perceived as personally relevant [24], impact on engagement and are difficult to optimise without experiential data from users. Gaining a better understanding of the user experience of behaviour change techniques is thus useful as it can help to provide insights into appropriate theory selection and operationalisation of behaviour change techniques.

Previous research on young women’s experience of smart phone apps for physical activity is limited. To the best of our knowledge there have been a few mixed gender qualitative studies focused on experiences of app use by university students (who tend to be young adults) [25, 26], and young adults [27] but not young women specifically. This prior research does suggest that young women have a unique perspective and needs compared to their male peers. For example, in one study where university students were presented with examples of app features in focus groups, female participants discussed safety concerns when presented with location sensing features [27]. The other studies provide insights into preferences and experiences of young adults, such as wanting feedback on activities, being able to set goals and monitor progress and not being interested in sharing workouts on social media [25, 26]. Whilst useful, the results of these studies are not specific to young women and focus more on preferences rather than an in-depth understanding of user experiences.

The present study aims to address gaps in knowledge about young women’s experiences with behaviour change techniques in physical activity apps by using qualitative, participatory research methods.

In particular, the study aimed to address the following research questions: 1) What are young women’s experiences of behaviour change techniques utilised in a popular smartphone app, and 2) are there factors that impact young women’s behaviour that are not addressed by a popular smartphone app? Qualitative research methods are well-placed to generate insights about factors that affect user experience compared with quantitative methods. This includes information about how techniques make users think and feel, reasons for non-engagement with specific techniques, and external factors that may impact users’ experience with apps [28].

## Methods

This study was conducted with approval from the University of Melbourne Psychology Health and Applied Sciences Human Ethics Sub-Committee (ID: 2056596). Methods and results are reported according to the American Psychological Association Journal Article Reporting Standards for Qualitative Research in Psychology [29] and the Standards for Reporting Qualitative Research [30].

### Study design

**Fig. 1:**
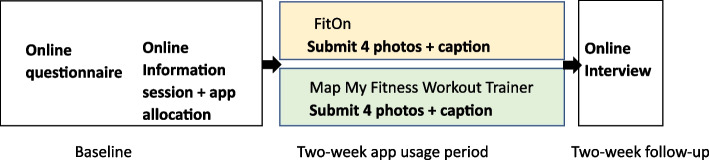
Study design

This study employs a participatory qualitative design, with participant asked to use one of two mobile applications for two weeks (randomly allocated), document their experience during those two weeks using photovoice techniques, and participate in a follow-up interview (see Fig. 1). The study was conducted online due to social distancing restrictions in response to the COVID-19 pandemic.

### Participants and recruitment

Participants were recruited online via paid Facebook advertising targeting young women nationally living anywhere in Australia from June–July 2020. Responding viewers (15,600 total views) were directed to an online eligibility questionnaire. Participants were eligible if they: lived in Australia; identified as female; were aged 18–24 years; owned a smartphone; did not meet physical activity guidelines [31]; and did not have a medical condition preventing them from completing physical activity. Eligible participants were prompted to complete an online consent form and were offered a $30 voucher as an incentive for study completion and their time.

### Physical activity app selection

Physical activity apps were identified from the health and fitness sections of Google Play and the Apple App Store (see Additional File 1 for inclusion criteria). Two apps were selected: FitOn and Map My Fitness Workout Trainer. Both had over one million downloads on Google Play, and an average user rating > 4.5 stars. These two apps were considered sufficient to enable participants to experience a range of common behaviour change techniques that could be compared across participants. Additional apps were not considered due to resource constraints. Additional File 2 summarises key features of both apps using the Coventry, Aberdeen, and London – Refined (CALO-RE) Taxonomy of Physical Activity Behaviour Change Techniques [32].

### Photovoice methodology

Photovoice is a participatory research method that empowers participants to identify experiences and concerns through photographs [33, 34]. By utilising photographs participants have taken, and then selected to discuss, discussions can be guided to reflect upon the reasons, emotions and experiences that guided participants chosen images. As participants documented experiences in real time and have a visual cue to reflect upon the ecological validity and richness of the findings is enhanced [35, 36].

Photovoice can be adapted along a participatory continuum to suit the research design and purpose, ranging from individual, photo-elicited discussions to group interviews that collectively generate themes [28]. For the current study, the researchers judged that individual interviews were more appropriate with participants using online data collection methods. In addition, using Photovoice online allowed the study to use a national sample with a population that the researchers had no prior relationship with, unlike traditional approaches to Photovoice that often rely on engaging local communities or groups through pre-existing relationships. Therefore, using Photovoice online gave the researchers the possibility to generate rich insights about young women’s experiences from a geographically diverse population that may not normally have been engaged in research [37]. Prior to the study the research team were not experienced with the Photovoice methodology.

### Procedure

Consenting participants were prompted to complete an online questionnaire, which collected demographic information, potential time barriers (i.e., study, work, and caring commitments) [38, 39], past experiences using physical activity apps, exercise motivation [40], and confidence to participate in regular physical activity [41]. Each participant attended an individual online information session (~ 20 min) and was randomly assigned (1:1 ratio) to a pre-selected smartphone app. Participants were instructed to use the app for two weeks to achieve a personal goal, but were not provided instructions on how to use the app. As previous research suggests that many who download apps disengage quickly, and non-usage attrition by two weeks is significant, a two-week period was considered satisfactory for this study [42].

During the two-week period, participants were instructed to use the ‘Photovoice’ technique to visually document their experience. Participants were provided prompts about what to consider photographing (e.g., facilitators, barriers, or perceptions). Photographs were not required to be specific to the app and could relate to the participants experience more broadly. Participants were instructed to send the researcher a minimum of four photographs accompanied by short descriptive caption.

After two weeks, participants attended a second, individual online interview with the researcher (40–45 min). A semi-structured guide was developed based on the SHOWeD technique (see Table 1), a series of prompts used to elicit open responses and help participants discuss the context and meaning of their photographs [33, 43]. Questions were adapted for individual use and to facilitate discussion of experiences that addressed the research aims. Interviews were recorded and transcribed using Otter.ai, and transcripts were manually compared against recordings to produce a verbatim transcription.

**Table 1:** Adapted photovoice questions from SHOWeD technique

Adapted Question	SHOWeD Technique
1. Can you describe your photo?	What do you See here?
2. What is happening in your picture?	What is really Happening here?
3. Why did you take a picture of this?	How does this relate to Our lives?
4. What was it about the app that led you to this experience?	Why does this situation, concern, or strength exist?
5. How did you think or feel about your goal because of this experience?	Why does this situation, concern, or strength exist?
6. What could we learn from this photo about how to improve physical activity supports for women like you?	What can we DO about it?

### Data analysis

The target sample size (*n* = 30) was based on data saturation considerations (i.e., sufficient to explore a range of contexts and perspectives in an in-depth way) [44]. Descriptive statistics were calculated for participant characteristics using questionnaire data. Analysis was conducted in NVivo 12 Plus. An inductive approach to thematic analysis was used to iteratively generate themes about women’s experiences using the apps from photographs and interviews [45], and an essentialist approach to analysis was used to develop findings from the data that directly reflected the individual participants’ experiences. This is in contrast with a constructivist approach that would seek to understand how larger sociocultural contexts influence individual experience [45].

An initial coding framework was developed identifying codes relevant to young women’s experiences of using the mobile application. The framework was independently applied by three researchers (MT, CS, YH) to both interview and photographic data for three participants. Coding was compared and discussed to clarify definitions and the coding approach. The coding framework was refined and applied by one researcher (MT). Sentences or phrases that meaningfully captured participant experiences formed the unit of analysis. A review of Photovoice methods in health and public health literature found that there is less information and consistency about the methods used to analyse photographic data [28]. Therefore, in keeping with this study’s essentialist approach to thematic analysis, the coding framework was applied to photographs as a whole, rather than undertaking a compositional analysis of the photograph, to directly capture participants’ key experiences. Codes were then collated into themes and subthemes and were iteratively reviewed and refined by the research team. Subthemes were included to capture the rich range of experiences related to an overarching theme. Both themes and subthemes were synthesised to address the research questions, namely how behaviour change techniques and external factors impacting physical activity were experienced by participants [45]. Conceptual frameworks were created for each theme to visually represent relationships between how the techniques were experienced and physical activity behaviour, including whether the technique was present or not, intervening factors that impacted participants’ experience, and the impact of a technique on the participant and their physical activity. The conceptual frameworks for each theme are presented in Additional File 3.

For each transcript, a conceptual diagram was created to map each participant’s experience, and diagrams were emailed to participants, with information on how to read the diagram, to confirm that it accurately reflected their experience and to seek additional input or correction [46]. Thirty participants confirmed they received the diagram, and none provided additional input. Diagrams were used to compare and validate conceptual frameworks for key behaviour change techniques across participants. Analytic memos were also used to document the researcher’s interpretations and insights about the raw data and to make meaning of the data by refining and identifying interrelationships between themes [47]. Analytic memos were created for each participant to link specific codes with any identified behavioural changes. These memos enhanced the dependability of the results by transparently documenting how the data were analysed and to synthesise key themes that clustered together [46].

## Results

Of those who responded to the ads (*n* = 124), 68 were eligible and provided consent. Of those, 39 scheduled an information session (*n* = 7 withdrew for personal reasons) and 32 completed the study (see Table 2 for participant characteristics). Seventeen participants were randomly assigned to FitOn and 15 to Map My Fitness. Participants set behaviour (e.g., frequency or type) and outcome goals (e.g., improved mood or weight loss) (see Additional File 4). Participants provided a total of 134 photos (Mean per participant = 4.2; Minimum = 3; Maximum = 8). Most participants were university students, who had prior experience using a physical activity app and moderate-high confidence for engaging in physical activity.

**Table 2:** Participant characteristics (*n* = 32)

Characteristic	n (%)
**Tertiary study**	
Domestic student	16 (50)
International student	9 (28)
Not a student	7 (22)
**Enrolment**	
Full-time	22 (69)
Part-time	3(9)
**Study type**	
Undergraduate program	15 (47)
Graduate program	10 (31)
**Employment**	
Not employed	15 (47)
Casual	11 (34)
Working part-time	5 (16)
Working full-time	1 (3)
**Caring responsibilities**	
No	32 (100)
**Experience using physical activity app**	
Never	10 (31)
Yes, I have in the past	19 (59)
Yes, I am currently using an app	3 (9)
**Duration of App Use**	
Less than 1 month	12 (38)
1–2 months	5 (16)
3–4 months	1 (3)
Longer than 6 months	4 (13)
**Exercise motivation**	
I feel guilty when I do not exercise	11 (34)
I value the benefits of exercise	17 (53)
I exercise because others say I should	2 (6)
I exercise because it is fun	2 (6)
**Confidence to participate in regular physical activity**	
A little confident	3 (9)
Moderately confident	11 (34)
Very confident	9 (28)
Extremely confident	9 (28)

### Thematic analysis

**Fig. 2:**
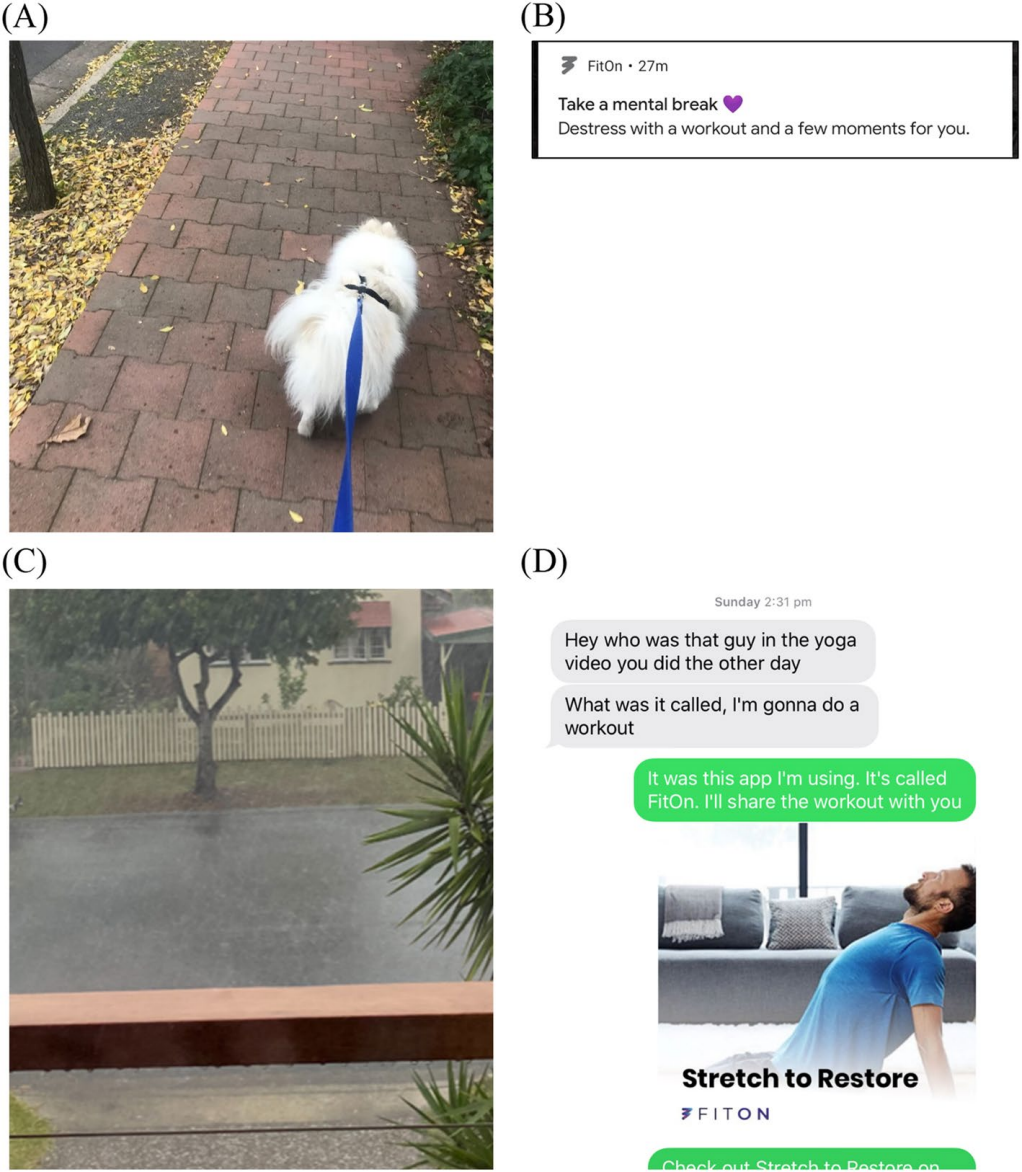
Illustrative photovoice data; Pictures promoted discussions on **A**) the participant’s reflection on mild activity undertaken after tracking dog walks; **B**) the type of notification that actually made them want to workout, **C**) how the app helped them to overcome an external barrier (bad weather) and **D**) social elements of using apps

Two central organising themes were utilised: mobile app techniques and external factors. User’s experiences with mobile app techniques clustered around four broad behaviour change technique categories, or themes: logging and monitoring physical activity; reminders and prompts; workout videos and written instructions; and social features. Social influences were also noted as a key external factors (see Table 3 for themes and subthemes). The results are outlined in subsequent sections and report key findings for each theme from the conceptual frameworks. Results from both apps are reported in combination, as sub-themes did not appear to be related to how the technique was operationalised within the app, but rather, if techniques were present and functional. Illustrative participant photographs are reported in Fig. 2 below.

**Table 3:** Summary of themes and sub-themes from photovoice and interview data

**Central organising theme 1**	**Mobile app techniques that influenced physical activity**
**Theme**	Logging and monitoring physical activity
**Subthemes:**	Fosters a sense of achievement
	Indicators should be personalised and meaningful
	Tracking has no effect on motivation for some users
	Inability to track is demotivating
**Theme**	Reminders and prompts
**Subthemes:**	Need to exercise
	Ineffective reminders were perceived differently
	Lack of reminders means no accountability
**Theme**	Workout videos and written instructions
**Subthemes:**	Improve knowledge and confidence
	Help overcome environmental barriers
	Trainers need to be relatable
	High intensity experiences lead to disengagement
	Both choice and guidance are important
**Theme**	Social features
**Subthemes:**	Potential to motivate but privacy concerns
	Young women uninterested in using apps with friends
**Central organising theme 2**	**External factors that influenced motivation and physical activity**
**Theme:**	Social features
**Subthemes:**	Friends and family positively motivate young women
	Groups are safe spaces to do exercise
	Social support is more effective outdoors

### Logging and monitoring physical activity

#### Fosters a sense of achievement

Participants discussed that logging and monitoring physical activity was important while working towards their goals. Users felt a sense of achievement and more capable of doing physical activity, with several discussing that monitoring served as external evidence of progressing towards their goal. Of note, many participants logged and monitored activity outside of their randomly assigned app such as using a smart watch, stopwatch, or other device to monitor physical activity, underscoring the importance of this feature as a facilitator. Other participants discussed that monitoring behaviours and outcomes helped support an intention to improve their performance.

*“... tracking the dog walks I go on every day could be a nice way to help me feel encouraged on the days I feel I haven’t been very active... being more intentional and aware of my more mild exercises made me feel like moderate exercise was more achievable and less... faraway. It really reminded me that ‘oh, I am actually doing something with my body every day’. And it wasn't such a huge jump from ‘I do nothing’ to ‘time to exercise all the time’.”* (Participant 08, Map My Fitness, see Fig. 2A)

*“I really like this feature about the app - how you can record the duration, calories... I think it's great for tracking and recording changes in my exercise routine... when you look back on those things that you did last, you want to try to better that like... because otherwise you just kind of like forget. And you don't try to strive for a bit of improvement.” (Participant 29, FitOn)*

#### Indicators should be personalised and meaningful

Motivation was dependent on whether participants perceived indicators to be meaningful and personalised. Performance indicators included measures of behaviours (e.g., number of workouts) and outcomes (e.g., calories). Several participants commented that indicators relating to physical appearance (e.g., calories/bodyweight) were not aligned with their goal and were not useful. In some cases, this led to disengagement. It was important for participants to be able to personalise and customise indicators according to their goal, or to display information that was personally motivating. Participants discussed that it was important for apps to offer a range of indicators and display information in a variety of ways to cater for young women, though no consensus emerged regarding specific indicators important for young women.

*“Yep. So this is um, I think the home page or like the home page of your profile. I think this page is sort of to motivate you. So it says like, how many workouts you’ve done, achievements... but at like the top and the focus of it is the calorie count. And so when you put in any workout you’ve done in the app... then it tells you how many calories that is. Um, and I feel, uh, that’s sooo triggering... your whole worth just becomes points.”* (Participant 01, FitOn)

#### Tracking has no effect on motivation for some users

Some participants found logging and monitoring had no effect, experiencing it as neither motivating nor demotivating, and not as a technique that encouraged them to do exercise.

*“I don't really care about what the numbers are... that's not my focus either... my goal for myself is just I want to exercise. I don't really care about like what I do.”* (Participant 40, Map My Fitness).

#### Inability to track activity is demotivating

Many participants were unable to track external activities, due to a range of issues including not finding or understanding how to log activities, inaccurate recording of data, or forgetting to use the app. This tended to result in participants disengaging, feeling discouraged, and in some cases, doing less physical activity. Participants commented that the inability to log their activities demotivated them because they felt that what they had done “did not count”. Therefore, tracking was motivating only if it accurately reflected all completed physical activity and progress towards their goals.

*“I was trying to think of a way to represent that I forgotten to use the app a few times. It made me a bit disappointed. My goal was to workout 30 minutes, if I'd missed the first five or 10, I was like, ‘ughh, well, now I'm gonna have to walk for more it's not gonna look like I've achieved when I actually did, because I started it later’. So it was a bit kind of like, ‘well, I might as well not even go on my full walk now because it’s not gonna show it in the end’.”* (Participant 30, Map My Fitness)

### Reminders and prompts

#### Need to exercise

Participants who used app or phone reminders (i.e., alarms) discussed that prompts made them feel they had to do physical activity and provided a form of external accountability. Several participants experienced reminders as a motivating or positive experience, with one participant discussing that this was due to encouraging language that emphasised the enjoyable aspects of exercise. Reminders prompted some to engage in physical activity, though for others, they created an expectation of performing physical activity, which did not necessarily lead to behaviour change, due to barriers (e.g., time).

*“I treat my fitness app like an accountability buddy... told the app to give me a notification at 2pm every day so that if I hadn't done it by 2pm, I then had to go and do it… Yep, I definitely think the accountability aspect... was a really big thing for me, because otherwise it was it felt like there was no one in checking on my progress. And in that case, I could just get away with not doing anything.”* (Participant 35, Map My Fitness)

*“This is the first workout related notification that actually made me want to work out. So it’s sort of um a gentle reminder working out, but also reminding you why it’s good to work out and that working out is for you, and not some kind of obligation... it highlights the positive side of this app. And something that I was actually quite surprised by... I actually didn’t end up working out for either of them but that is also due to having like, studies and not enough time. But they made me feel, more like, motivated, to work out.”* (Participant 01, FitOn, see Fig. 2B)

#### Ineffective reminders were perceived differently

A few participants discussed that reminders were not effective at prompting exercise, due to time barriers or low motivation. When reminders were ineffective, some participants did not perceive them negatively, and expressed that they still had the potential to provide external accountability. Other participants experienced guilt, which did not prompt them to overcome perceived barriers to physical activity.

*“The thing the thing about reminders for me is that it gives me a little prompt, but if I don't feel in the mood, to do whatever is prompting me to do I just swipe it off and I can easily forget about it and not really feel that guilty about not doing something.” (Participant 31, FitOn)*

*“Because I had set up reminders... I used to look at that. And then I used to feel guilty that I'm not using the app when I should be exercising. And then I just turned them off, so I stop feeling bad about myself (*laughed*).”* (Participant 07, FitOn)

#### Lack of reminders means no accountability

Several participants were unable to use reminders due to: reminders not working, participants not knowing they were a feature, or the app not having reminders. Map My Fitness removed reminders from the free app available in Australia during 2020, resulting in some participants not having access to this feature. When app reminders did not work, participants tended to forget to do planned exercise and disengaged from the app.

*“So like, because the app didn't remind me in the first week... by, you know, day seven, day eight, [it] kind of faded and was the app wasn't supporting that. I kind of just, forgot about it.”* (Participant 30, Map My Fitness)

External accountability was highlighted as being important for participants, as having a goal was not sufficient to overcome low motivation to exercise. A few participants expressed that reminders would have helped them achieve their goal if they had used the technique. However, participants also highlighted that reminders may not have been sufficient to overcome low motivation, in part due to the app lacking in-person accountability or consequences for non-compliance.

*“The motivation was probably the one that I think over time that would probably be an issue for me. Like not having someone there to prompt me would probably mean I'd not do the videos and eventually just give up... I think I would get to the point that I would ignore the reminders.”* (Participant 04, FitOn)

### Workout videos and written workouts

#### Improve knowledge and confidence

Workout videos were important for improving knowledge, confidence, and motivation to try new or unfamiliar activities, particularly among those less experienced. Several participants discussed that demonstrations would have helped them feel they were performing exercises effectively, while others discussed that performing exercises along with a video demonstration provided a form of motivation.

*“… because I’m new to exercise I try and to learn from the videos. So I enjoy it. And I used it mainly for education, meaning that I learn the moves... I do not know there's so many stretch, so many ways to do stretch and to do yoga and there's also something called, pilates. It actually taught me many things I did not know before.”* (Participant 18, FitOn)

#### Help overcome environmental barriers

Videos and written workouts helped overcome environmental barriers, (e.g., weather, time of day, lack of space or equipment), which was particularly important for users experiencing coronavirus restrictions. Interestingly, although participants discussed that structured app workouts facilitated exercise, they often enjoyed their typical activities more. Only one participant identified that videos changed her attitude to exercise and that she enjoyed exercise more than she had previously.

*“So that is a photo of the rain outside. Which, I guess, is a barrier that happens with me occasionally here. So I would normally go and do exercise outside. However, on particular days where it's raining, I just wouldn't do it usually wouldn't do anything and just kind of skip it. However, with the app, I could do indoor exercises, obviously, and ones in smaller spaces, which I found a much better alternative to doing nothing.”* (Participant 12, FitOn, see Fig. 2C)

#### Trainers need to be relatable

The relatability and diversity of trainers affected participants’ engagement. Users either felt motivated or demotivated depending on how relatable they perceived trainers to be. Participants primarily discussed that the range of body types, ethnicities, and genders affected their motivation. Many perceived trainers as unrelatable or felt they were not engaging. Participants who perceived themselves as having lower levels fitness levels, discussed that trainers made them feel that the exercises may be unachievable. Others perceived that the trainers had diverse body types and genders, which promoted confidence. One user discussed that the positive language trainers used was encouraging for beginners and helped her develop the expectation that exercise would improve her physical appearance.

*“I was really put off by the first impressions of the app... like, when you're not very fit, it's always super intimidating to look at people or videos of people who are really fit. And then to think, I'm just like, how can I achieve that when I'm, you know, so much at a lower level. But, I think, I know that most of that is in my head...”* (Participant 06, FitOn)

*“As well as seeing there were there were three trainers in the video and they will have different body sizes. And even the one that looked she was a larger woman... and seeing her be able to keep up with whatever the other two are doing... felt like, if she can do it, so can I. Yeah, it felt, more motivating seeing a male trainer do it as well.”* (Participant 31, FitOn)

#### High intensity experiences lead to disengagement

Many participants disengaged after experiencing high intensity exercise, discussing that they felt demotivated when they could not physically complete the exercise or if the exercise was more difficult than anticipated. Low-intensity exercise options were important for participants to feel that they could achieve their goals. However, after a high intensity exercise experience, most participants disengaged from the app instead of searching for lower-intensity exercises.

*“I think because I did try to do a few of the exercises. And just like maybe a few minutes in, I would just feel so horrible and demotivated, I guess just cuz, like some sort of exercises... like I just physically couldn't do and it just made me feel sad, I guess... I just like, looked down and I just thought, ‘Oh, this will kind of share how I'm feeling just kind of demotivated and down, I guess’.”* (Participant 33, FitOn).

#### Choice and guidance are important

Participants discussed that exercise choices and directive guidance were equally important to feel that physical activity was achievable. Some participants discussed that the variety of videos helped them feel they had control over their physical activity and could find exercise that suited their lifestyle and preferences. Both apps included video or written workouts that varied by exercise type, length, equipment, and circumstances (e.g., small living spaces). While variety was helpful, participants discussed that the workouts did not provide guidance on how to exercise effectively or structure a program of physical activity. This made participants feel that the videos or written workouts were not helpful for them to achieve a goal and led to disengagement.

*“So I could kind of do what I was feeling like because sometimes I'm more into the fast paced kind of workouts and sometimes yoga and things is more suited at whatever time I'm working out. So I found the range and variety a nice benefit of the app. Yeah, um, the different varieties makes me think, because I can maybe exercise more frequently. Because if I don't have time for, or like, I'm inside or I may have a small space, those kind of things. It makes it more accessible.”* (Participant 12, FitOn).

*“One of the barriers that I've always found when it comes to working out is that like, I don't really know what to do in terms of, like it's very easy to start running. But then like, are you supposed to do it in a certain way?... Like, there has to be some like, I guess, science to it... so it helps you achieve a goal. But yeah, like I guess that comes into like giving you direction. I found that these apps don't really do that.”* (Participant 34, Map My Fitness)

### Social features

#### Potential to motivate but privacy concerns

Four participants engaged with social features, which aimed to facilitate social comparisons (e.g., activity feed) and social support (e.g., invite friends to use the app). These participants reported that it enhanced their motivation, such as increasing feelings of relatedness with friends also using the app. Others expressed positive views of social features but had not engaged with them. Two participants discussed privacy concerns on Map My Fitness, which led them to delete the app. One user found that the app displayed the GPS walking routes of her neighbour, while another received a friend request through the app for a dating website. These participants discussed that social features should be designed on an opt-in basis to prioritise the safety of female users.

*“I guess it's sort of also, you have a bit more like accountability, if you know, the people around you are also using the app... having like my boyfriend or my friend, like, not necessarily push me but be like, “Oh, I'm going to do this workout.” And then that would sort of motivate me to do it as well or work out with them... that was pretty much the only thing that would have had motivated me to actually do it.”* (Participant 32, FitOn, see Fig. 2D)

*“But then when I clicked into it, it came up with the route and then my neighbour's name. And I was like, ‘Oh my god.’ Like this was done like as you can see, like 2013, years and years ago. Still sitting there with her full name and the start and end of her walk and I just think like I would never walk on my own in the dark, even though I live in a safe neighbourhood. I always do, just check behind me to see if anyone's following when I'm out on my own. And so knowing there’s like data, and mine would be tracked too, that anyone else could access, just freaked me out. I didn't like that.”* (Participant 30, Map My Fitness)

#### Young women uninterested in using apps with friends

Participants did not engage with social features for several reasons, including being uninterested in other’s physical activity, concerns that friends would negatively assess their performance, and perceiving that sharing physical activity information was not the norm among peers.

*“Like I think this app is supposed to be a little bit social and that you have friends and stuff. Like, I guess I'm like young and like in my friend group, we, like this is not a thing. Like you don't share your workouts with people, it's kind of weird.* (Participant 34, Map My Fitness)

#### External social features

Three social factors emerged, external to the apps: (1) friends and family positively motivated young women, (2) groups are safe spaces to do physical activity, and (3) social support is more effective outdoors. Many participants involved friends and family in their physical activity, which was a key strategy that motivated participants to exercise and enhanced their enjoyment. Exercising with people they knew helped participants feel that someone would positively motivate them, provided a forum to share their successes and challenges, and was a way to learn exercise from a trusted person. Exercising as part of a group also facilitated exercise by helping participants feel less self-conscious about their physical abilities and created a safe space where they could relate to others. Doing physical activity with friends and family outdoors was discussed by many participants as a factor that enhanced their enjoyment and motivated them to work towards their goals. Participants identified that engaging social support outdoors was more fun, relieved stress, and improved their mental health. Many participants discussed that their mental health and being outdoors was equally as important as physical activity, which may have been further emphasised due to coronavirus restrictions.

*“Well, I definitely feel more comfortable like exercising with a friend, than exercising in a gym. Like even going to a group exercise class in a gym is still better than going alone. But for a person like me who's not super fit, it's still very intimidating. So yeah, it's like a very safe space.”* (Participant 06, did workouts with friends over Zoom)

*“I guess going for walk on the beach is, that is exercise. And I guess, one of the things that I really I really enjoyed about was I was kinda helped my mind a little bit as well, that was going for a walk on the beach. I was kinda switching my head off.”* (Participant 04, FitOn)

## Discussion

This study explored young women’s lived experience of behaviour change techniques employed in popular physical activity apps. In doing so it has generated valuable insights into the process through which common behaviour change techniques may influence physical activity behaviour in this cohort, and highlighted important considerations for operationalising behaviour change techniques effectively to promote both engagement and behaviour change.

Promisingly, participants’ experiences in this study suggest the behaviour change techniques explored can help to change important determinants like motivation and competence, as outlined in prominent behaviour change frameworks (e.g., the behaviour change wheel [48]) and commonly applied theories in mHealth interventions (e.g., social cognitive and self-determination theory [23, 49]). Self-monitoring, for example, appeared to positively impact goal setting and feelings of competence, with some participants reporting that it led to them feeling more capable and setting higher goals. What participants were able to log, however, seemed to be an important influence on their experience. If exercise could not be logged, participants perceived that their exercise was less impactful or "didn't count”. The ability to log or sync activities that are common among young women is recommended [50]. Further, indicators relating to physical appearance (e.g., calories/weight) were described as “triggering”. These results align with previous research suggesting that indicators should be personalised to individual goals [51] and that monitoring may lead to unhealthy fixations [52]. These results add to the literature by identifying potential features that can negatively influence young women’s behaviour change experiences. App designers should enable users to opt-in to indicators, as not all indicators are equally helpful. Indicators that focus on behaviours, rather than outcomes (e.g., weight) may be most beneficial, and could help to foster more intrinsic forms of motivation [53, 54].

Videos and written workouts also had a positive impact on competence for some participants by helping them believe they were capable of doing new exercises. Having a variety of exercises to choose from gave some participants a sense of control, and enhanced opportunities for home-based exercise during coronavirus lockdowns. Conversely, not having access to videos, having no guidance about which exercises to choose and/or the exercises in the videos being too difficult led to participants feeling less confident. For this technique to be effective it is recommended that easier workouts are included so that users can gain confidence through feelings of mastery. The relatability of trainers also seems to be an important factor for operationalising this technique, however participants experienced video trainers differently. It is unclear why participants with seemingly similar characteristics (e.g., ethnicities, fitness levels) responded differently. This unexpected result suggests that other determinants or individual-level traits may moderate the effect that videos have on user self-efficacy. A potential improvement would be to include diverse trainer-types to account for individual differences. However, future research is needed on what factors impact perceived relatability, and how or when this is likely to impact engagement.

Reminders helped participants focus attention on physical activity goals (in line with prior research) [23, 55]. When reminders did not work within the app, several users forget to exercise, suggesting they needed external support to focus attention on their goals. Reminders also had an impact on motivation type. One user explained that the reminder’s emphasis on stress-relief helped her feel that physical activity was important for self-care and increased her motivation to exercise. Conversely, one participant discussed that reminders made her feel guilty when she did not exercise leading to disengagement. Drawing on self-determination theory, and previous studies in the general population [51, 52, 56–58], it may be that reminders are more likely to be effective when they promote identified (i.e., valuing physical activity as personally important) or intrinsic motivation (inherent satisfaction, enjoyment) [53, 54], and promote disengagement when they promote introjected motivation (e.g., guilt, shame).

In contrast to other key techniques, in-app social features were not discussed as being as helpful for physical activity behaviour change. Apps included a range of social features aimed at positively targeting user’s self-efficacy, relatedness with others, and user subjective norms. Participants frequently discussed reasons for not engaging, including not feeling motivated by other users’ physical activity. As seen in a British study with university students [27], some participants also identified safety concerns with apps sharing personal information. Notably, young women in Australia have an increased risk of experiencing online sexual harassment through phones, social media, and dating apps [59]. Previous research shows mixed results for the association between social features, app engagement, and physical activity. In particular, studies report different user preferences [51, 52, 60], which may be due to the range of social comparisons that apps facilitate [61]. Previous research also suggests that experiences may be moderated by individual-level traits, such as trait-competitiveness, age, and gender [62, 63]. Therefore, more research is needed to examine the influence of individual-level traits and identify on social features that may be appropriate for different clusters of inactive young women. However, external social support appeared to positively influence participant motivation and behaviour. Compared to online social support with strangers, drawing on their own networks may feel safer and more satisfying for young women. Future research should aim to determine if and how techniques to facilitate social support, including from existing networks, can be better incorporated into apps for young women.

The strengths and limitations of this study should be considered when generalising and building on these results. The semi-longitudinal design and use of photovoice methods enhance the ecological validity of the findings, by gathering data about participants’ actual behaviour change processes. The adequacy of the data was also enhanced by the number of participants nationally recruited, and the variety of participant data collected. The trustworthiness of results was improved by conducting member-checks, and using analytical memos, participant flow-diagrams, and conceptual frameworks. However, participants were predominantly university students, and experiences may have been influenced by this shared characteristic. Further, several participants discussed that their participation served as motivation to engage with the app, or to work towards their goal. Whilst participant experiences may have been influenced by study participation, the use of a control group was not possible with photovoice methodology. Finally, the qualitative methods did not allow for analysis of individual characteristics that may have influenced participants’ experiences and we did not collect quantitative engagement data with the apps.

## Conclusion

This study highlights important implications for optimising physical activity apps for young women. Noteworthily, young women’s lived experiences of commonly used behaviour change techniques suggest they are promising for influencing important determinants of behaviour change, such as motivation and competence. However, there are issues that need to be addressed, such as the operationalisation of techniques being triggering (in the context of body image), inducing guilt, or leading to privacy or safety violations. Future research focused on overcoming these limitations is recommended.

## Supplementary Information

**Additional file 1.** Summary of inclusion criteria for app selection.

**Additional file 2.** Summary of key behaviour change techniques and features in selected physical activity apps.

**Additional file 3.** Conceptual frameworks.

**Additional file 4.** Individual participant goals.

## Data Availability

The datasets used and/or analysed during the current study are available from the corresponding author on reasonable request.

## Abbreviations

Apps: Applications
CALO-RE: The Coventry, Aberdeen, and London – Refined Taxonomy of Physical Activity Behaviour Change Techniques
