# Supplementary material for: Using photovoice to explore young women’s experiences of behaviour change techniques in physical activity mobile apps
Source: Int J Behav Nutr Phys Act. 2023 Apr 14;20:43. doi: 10.1186/s12966-023-01447-9 (PMC10101820; doi:10.1186/s12966-023-01447-9)
Supplement: Supplementary file 1 — Additional file 1. Summary of inclusion criteria for app selection. [file 12966_2023_1447_MOESM1_ESM.docx]

**Additional File 1****.** Summary of Inclusion Criteria for App Selection

| **Inclusion Criteria** | **Description** |
| --- | --- |
| **Content** | Promoted physical activity (other health and wellbeing apps were excluded, e.g., calorie-counters, meditation, sleep); varied in the type of behaviour change techniques included; and reflective of common apps that Australian women report using for fitness (38), to increase ecological validity of the results. |
| **Users** | Popular and widely used (determined by user rating and number of downloads); exclusively targeted to women, or to both women and men. |
| **Availability** | Free for ongoing use; no purchase of an additional device required (can include ads and in-app purchases); available on both Android and iOS devices. |
