# Supplementary material for: Using photovoice to explore young women’s experiences of behaviour change techniques in physical activity mobile apps
Source: Int J Behav Nutr Phys Act. 2023 Apr 14;20:43. doi: 10.1186/s12966-023-01447-9 (PMC10101820; doi:10.1186/s12966-023-01447-9)
Supplement: Supplementary file 2 — Additional file 2. Summary of key behaviour change techniques and features in selected physical activity apps. [file 12966_2023_1447_MOESM2_ESM.docx]

**Additional File 2*.*** Summary of Key Behaviour Change Techniques and Features in Selected Physical Activity Apps

| **Behaviour Change Technique** | **FitOn** | **Map My Fitness** |
| --- | --- | --- |
|  | A variety of exercise videos with fitness trainers. Can personalise fitness goals and training plans. Includes social features (leader board) for exercise videos, workout challenges, and ability to invite friends to complete exercise videos. | Tracks routes, distance. and pace of physical activity via GPS. Contains written workouts, ability to create individual training plans. Includes social features (community feed), workout challenges, and ability to share workouts. |
| **Goal-setting (behaviour)** | Goal-setter: set duration of goal, workout frequency, duration, and type of workout video. | Goal-setter: set goal type (i.e., distance or duration) workout frequency and activity type (i.e., walking, running, cycling). |
| **Goal-setting (outcome)** | Goal-setter: set outcome to achieve through physical activity (i.e., increase activity, lose weight, tone, build muscle, reduce stress). | **—** |
| **Prompt self-monitoring (behaviour)** | Log activity: automatically logs time watched for exercise videos; list of external activities to log date, duration, and calories completed. | Log activity: GPS tracker (visual maps); list of external activities to log date, GPS route, distance, duration, calories, heart rate, pace. |
| **Prompt self-monitoring (outcome)** | Monitoring dashboard: displays behavioral outcomes (i.e., calories, completed) and calendar (day workout completed). | Monitoring dashboard: displays behavioral outcomes (i.e., distance, duration, calories), weekly chart of distance/day, and donut chart of progress toward weekly goal. |
| **Action planning** | Workout scheduler: Schedule workouts per week, length of workout, and workout videos. | Training plan: Schedule activity, date, and time, start and end dates. |
| **Teach to use prompts/cues** | Reminders: Schedule day and time of reminders; schedule app curated workout reminders for morning, afternoon or evening. | Reminders ^a^: Schedule day and time for training plan reminders within the training plan feature (see row above). |
| **Model/demonstrate the behaviour** | Video workouts: varying lengths, intensities, organised by exercise type or other category (e.g. small space, no equipment, beginner). Different trainers provide verbal instruction and visual demonstrations throughout. | List of individual exercise names to perform within the workout routine (e.g. calf raise, runner stretch). ^b^ |
| **Facilitate social comparison** | Video workout leader board: Number of users completing workout video at a time and shows user rank in terms of calories burned.  Group classes: Scheduled classes with users for specified day/time. | Activity feed: Community feed where users can share photos, comments, and statistics of completed workouts and other users can react and post comments. |
| **Facilitate social comparison** | Global challenges: Set challenges for all users to complete specified activities within a specified time.  Personal challenge: Select videos, date and duration with invited friends. | Global challenges: Set challenges for users to complete specified activities within a specified time. Displays rank against other users.  Personal challenge: Create challenge type (e.g. calories, distance) for specified activity (e.g. walk), date and duration with invited friends. |
| **Plan social support/social change** | Invite friends: Search for and invite friends to use the app.  Chat: Chat with friends in the app.  Schedule workouts with friends: Invite friends to complete video workout for scheduled day and time. | Invite friends: Search for and invite friends to use the app.  Share details: User can share completed workouts, routines, and routes with friends or all users. |

^a^ Reminders were removed from the free version of the app (in Australia) sometime in 2020, with some participants having access to reminders during their participation and others not having access.

^b^ Video demonstrations were unavailable in the Australian free version of the app but were available on the Under Armour® YouTube channel. Video demonstrations were added to some workout routines within the app sometime in 2020.
