# Supplementary material for: Using photovoice to explore young women’s experiences of behaviour change techniques in physical activity mobile apps
Source: Int J Behav Nutr Phys Act. 2023 Apr 14;20:43. doi: 10.1186/s12966-023-01447-9 (PMC10101820; doi:10.1186/s12966-023-01447-9)
Supplement: Supplementary file 3 — Additional file 3. Conceptual frameworks. [file 12966_2023_1447_MOESM3_ESM.docx]

Additional File 3. Conceptual Frameworks

Figure 1. *Conceptual Framework of Participants’ Experiences Logging and Monitoring Their Physical Activity*

Indicators are personalised and meaningful

Logging and monitoring physical activity

Able to log

Unable to log

Fosters sense of achievement

Belief physical activity is achievable

Intention to improve physical activity

Disengaged from app

Demotivation and discouragement

Yes

No

**+**

**More physical activity**

**─**

**Less physical activity**

Relationship shown by data

Relationship hypothesised but not shown by data

Figure 2. *Conceptual Framework of Participants’ Experiences Using Reminders and Prompts for Physical Activity*

Time barriers

Reminders and prompts

Present

Not present /

not working

**+**

**More physical activity**

Relationship shown by data

**─**

**Less physical activity**

Low motivation

Needs to exercise

External accountability

Motivated to exercise

Ignores reminders

Guilt

Forgets to exercise

Figure 3. *Conceptual Framework of Participants’ Experiences Using Videos and Written Workouts for Physical Activity*

Exercise is accessible

Doesn’t know where to start

Videos and written workouts

Present

Not present

Relationship shown by data

Improve knowledge and confidence

Overcome environmental barriers

Motivating

Exercise is unachievable

Not confident that working out effectively

Trainers

Intensity

Choice

**+**

**More physical activity**

**Disengaged from app**

Figure 4. *Conceptual Framework of Participants’ Experiences Using App Social Features and External Social Supports for Physical Activity*

Privacy concerns

App social features

Motivating

Feels accountable

Disengaged from app

**+**

**More physical activity**

Relationship shown by data

External social support (family, friends)

Uninterested in social features

Motivating and encouraging

Safe space

Feels part of a group

Enhanced enjoyment outdoors

**+**

**More physical activity**
