# Supplementary material for: Using photovoice to explore young women’s experiences of behaviour change techniques in physical activity mobile apps
Source: Int J Behav Nutr Phys Act. 2023 Apr 14;20:43. doi: 10.1186/s12966-023-01447-9 (PMC10101820; doi:10.1186/s12966-023-01447-9)
Supplement: Supplementary file 4 — Additional file 4. Individual participant goals. [file 12966_2023_1447_MOESM4_ESM.docx]

Additional File 4. Individual Participant Goals

| **Fit On** | | **Map My Fitness** | |
| --- | --- | --- | --- |
| **Participant number** | **Behavioural Goal** | **Participant number** | **Behavioural Goal** |
| 4 | To develop a structured routine when it comes to exercise | 2 | Go out for a walk every day for half an hour |
| 12 | To exercise on a more regular basis and/or exercise a certain amount of times per week | 3 | Take a 1-hour stroll or run outdoors at least 2 times a week |
| 14 | To be more active | 8 | To complete half an hour of moderate-intensity exercise 5 times over the next two weeks |
| 18 | To do stretching 30 minutes 3 times each week | 9 | 150 minutes of some outdoor exercising per week |
| 20 | To exercise for 1 hour, 3 times per week | 15 | To do 3-4 sessions of physical activity per week |
| 29 | To complete 3-4 workouts per week (15 minutes each, doing cardio-based exercise, such as running or walking on the treadmill) | 19 | To complete a 15 minute workout every day |
| 31 | To build a healthy habit and workout at least once per week up to 3 to four times per week | 21 | To hold a plank for 2 minutes |
| 33 | To get a regular physical activity routine happening over the next two weeks. Selected in the app to reduce stress as goal | 30 | To complete 30 minutes of exercise per day over the next two weeks |
| 39 | To do some physical activity workout for 10 to 20 minutes per day | 35 | To run 4 Km in under 24 minutes. To improve my flexibility generally, but in particular for my legs and lower back |
|  |  | 37 | To do my assigned sports program 3 times per week |
|  |  | 40 | To do 150 minutes of moderate intensity exercise per week |
| **Participant number** | **Behavioural Outcome Goal** | **Participant number** | **Behavioural Outcome Goal** |
| 1 | To have a full workout without feeling embarrassed or doubting myself | 5 | To lose weight |
| 6 | Work on my arm strength | 23 | To see the positive effects of exercise on my mood and energy |
| 7 | I want to be more fit | 26 | To use workouts on the app to improve my leg strength, and to run 3 km in under 20 minutes |
| 16 | To lose 1 kg in two weeks | 34 | To start running and increase my stamina/train for distance |
| 27 | To reduce my stress by exercising for 15-20 minutes, 3 to 4 times per week |  |  |
| 32 | To become more flexible |  |  |
| 36 | To lose 2 kg in two weeks |  |  |
